# Author Correction: Effective screening of Coulomb repulsions in water accelerates reactions of like-charged compounds by orders of magnitude

**DOI:** 10.1038/s41467-022-34650-6

**Published:** 2022-11-09

**Authors:** Adam Kowalski, Krzysztof Bielec, Grzegorz Bubak, Pawel J. Żuk, Maciej Czajkowski, Volodymyr Sashuk, Wilhelm T. S. Huck, Jan M. Antosiewicz, Robert Holyst

**Affiliations:** 1 Institute of Physical Chemistry, Polish Academy of Sciences, Warsaw, 01-224 Poland; 2 University of Zurich, Department of Chemistry, Zurich, CH-8057 Switzerland; 3 Lancaster University, Department of Physics, Lancaster, LA1 4YB UK; 4 University of Oxford, Department of Chemistry, Oxford, OX1 3TA UK; 5 Institute for Molecules and Materials, Radboud University, Nijmegen, 6525 AJ Netherlands; 6 Institute of Experimental Physics, Biophysics Division, University of Warsaw, Warsaw, 02-093 Poland

**Keywords:** Reaction kinetics and dynamics, Catalysis

Correction to: *Nature Communications* 10.1038/s41467-022-34182-z, published online 28 October 2022

In this article the funding from ‘European Union’s Horizon 2020 research and innovation program’ and ‘Minister of Science and Higher Education’ was omitted. The original article has been corrected.

