# Supplementary material for: Effective screening of Coulomb repulsions in water accelerates reactions of like-charged compounds by orders of magnitude
Source: Nat Commun. 2022 Oct 28;13:6451. doi: 10.1038/s41467-022-34182-z (PMC9616817; doi:10.1038/s41467-022-34182-z)
Supplement: Supplementary file 1 — Supplementary Information [file 41467_2022_34182_MOESM1_ESM.pdf]

Supplementary information for:
Effective screening of Coulomb repulsions in water accelerates reactions of like-charged compounds by orders of magnitude

Adam Kowalski,[1,†] Krzysztof Bielec,[2,†] Grzegorz Bubak,[1,†] Pawel J. Zuk,[1,3] Maciej Czajkowski,[4] Volodymyr Sashuk,[*,1] Wilhelm T.S. Huck,[*,5] Jan M. Antosiewicz,[*,6] Robert Hołyst[*,1]

1 Institute of Physical Chemistry, Polish Academy of Sciences, Kasprzaka 44/52, 01-224 Warsaw, Poland
2 University of Zurich, Department of Chemistry, 8057 Zurich, Switzerland
3 Lancaster University, Department of Physics, Lancaster, LA1 4YB, United Kingdom
4 University of Oxford, Department of Chemistry, Oxford, OX1 3TA, United Kingdom
5 Institute for Molecules and Materials, Radboud University, Nijmegen 6525 AJ, Netherlands
6 Biophysics Division, Institute of Experimental Physics, Faculty of Physics, University of Warsaw, 02-093 Warsaw, Poland
† These authors contributed equally.

* vsashuk@ichf.edu.pl, * w.huck@science.ru.nl, * jantosi@fuw.edu.pl, * rholyst@ichf.edu.pl

Supplementary Note 1. UV-VIS absorption and emission spectra.
We measured UV-VIS absorption and emission spectra of reactants and product in the presence of polylysine and cetrimonium chloride, see Supplementary Figure **1**. Steady-state absorption spectra were recorded with a Shimadzu UV-2401PC spectrometer, using a quartz cuvette (Hellma, Germany) with a 10 mm path length. Fluorescence spectra were registered using a Perkin-Elmer LS55 spectrofluorometer, using a quartz cuvette (Hellma, Germany) with a path length of 10 mm for excitation and 10 mm for emission. Both CoA-M and product have maximum absorption at $\sim$ 370 nm wavelength. However, they also show a signal at 500 nm wavelength. Thus, both 485 nm laser and 370 nm light-emitting diode used in different techniques were efficient to excite samples.

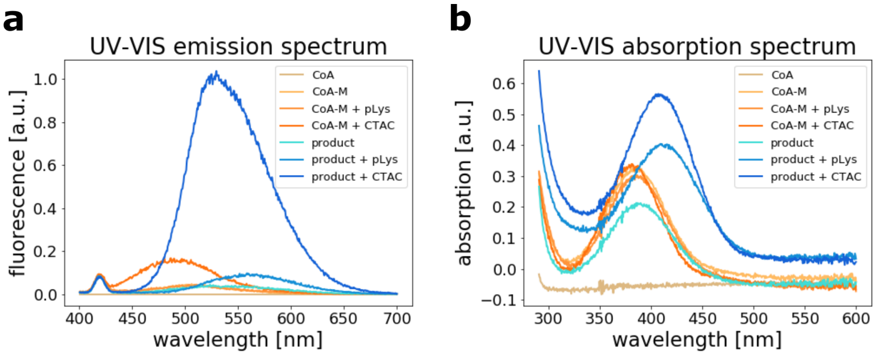

Supplementary Figure 1: **(a)** UV-VIS absorption and **(b)** emission spectra of reactants and product in the presence of polylysine and cetrimonium chloride.

Supplementary Note 2. Countrate of reactants and product for CoA and CoA-M reaction. We examined how fluorescence intensity varies with the change in reactants or product concentration in water, see Supplementary Figure **2**a. There is no change in the number of photons registered in time (countrate) for CoA since it is nonfluorescent. However, by increasing the concentration of CoA-M or product, one can observe linear growth of the countrate. This result is expected. While the more fluorescent molecules are in the solution, the more will be excited, and the more fluorescence will be registered.

Later, we calculated the tilt (tanα) of fluorescence intensity growth versus the concentration of CoAM or product with a linear regression model. Tanα for the product is $\sim$11 times greater than the reactant's one because of the change in quantum yield of the fluorescent moiety. We performed similar experiments in the presence of considered catalysts, as depicted in Supplementary Figure **2**b. Nona-arginine and polylysine slightly affect the fluorescent moiety properties, while CTAC increases its quantum yield by $\sim$3 folds. These changes suggest complex formation between reactant and catalyst. We also showed that the salt does not change the photophysical properties of the product, which shows Supplementary Figure **2**c.

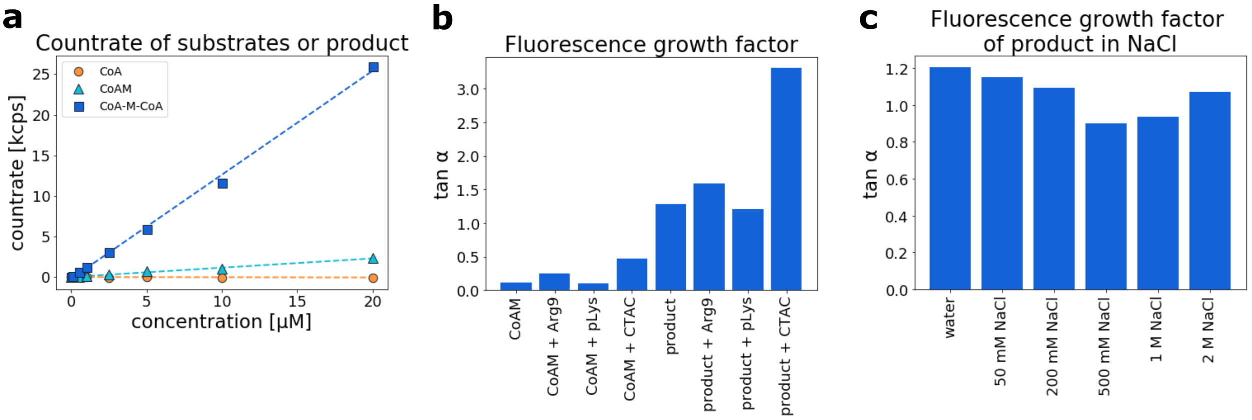

Supplementary Figure 2: **(a)** Change in the countrate of reactants or product with their change in the concentration in water, **(b)** tilt values for fluorescence intensity change versus CoA-M or product concentration in the presence of examined catalysts, **(c)** tilt values for fluorescence intensity change versus product concentration in various NaCl solutions. T = 25 °C.

Supplementary Note 3. Assumed catalytic model and error analysis.
The product, P, formation of CoA and CoA-M is an irreversible second-order reaction that can be described as the reaction between reactants A and B (Equation **1**).

$$\mathrm{A + B \xrightarrow{k} P} \tag{1}$$

By introducing to such a system catalyst C, the process becomes more complicated, as intermediates of reactant-catalyst occur, which depicts Equation **2**.

$$\mathrm{A + B + C \underset{k_{1-}}{\overset{k_{1+}}{\rightleftharpoons}} AC + B \underset{k_{2-}}{\overset{k_{2+}}{\rightleftharpoons}} BC + A \xrightarrow{k_3} P + C} \tag{2}$$

If the surfactants above CMC are catalysts, the reaction path has to be considered as in Equation **3**, which applies micelle ,D , formation and disintegration.

$$\begin{array}{c} \mathrm{A + B + C \underset{k_{1-}}{\overset{k_{1+}}{\rightleftharpoons}} AC + B \underset{k_{2-}}{\overset{k_{2+}}{\rightleftharpoons}} BC + A \xrightarrow{k_3} P + C} \\ \updownarrow \qquad \updownarrow \qquad\qquad \updownarrow \qquad\qquad \updownarrow \\ \mathrm{A + B + D \underset{k_{4-}}{\overset{k_{4+}}{\rightleftharpoons}} AD + B \underset{k_{5-}}{\overset{k_{5+}}{\rightleftharpoons}} BD + A \xrightarrow{k_6} P + D} \end{array} \tag{3}$$

However, we assumed that in the studied system (negatively charged reactants, positively charged micelles as catalysts) the formation and disintegration of micelles, as well as the association of catalyst with reactants do not significantly influence the rate order of the primary reaction. Catalysts do not affect the chemical reaction of CoA-M and CoA, and do not lead to another product formation. The catalysts are only utilized as attracting molecules for the reactants to meet each other at their surfaces. Therefore these reversible processes were neglected in the kinetic model of the reaction.

In our error analysis, we identify the most significant uncertainty is due to roughly 10% error of identifying concentration via light intensity measurements $[C] = [I] \pm 10\%[I]$. If we apply the error propagation rule to Equation 4 (Manuscript) and find that each measurement has the following measurement uncertainty

$$\Delta k_{I_t} = \frac{1}{t(I_f - I_t)^2} \times 0.1 I_t \tag{4}$$

due to I_t and

$$\Delta k_{I_f} = \frac{I_t(I_t - 2I_f)}{I_f^2 t(I_f - I_t)^2} \times 0.1 I_f \tag{5}$$

due to I_f. As a result error for each time instance t is given by

$$\Delta k_t = \sqrt{(\Delta k_{I_t})^2 + (\Delta k_{I_f})^2}. \tag{6}$$

So for each data point we find

$$k_t \pm \Delta k_t. \tag{7}$$

From those points, we can get the average value of k, considering uncertainty in each measurement through the weighted mean.

Supplementary Note 4. Coenzyme A - reaction in water.
The extremely slow kinetics of the uncatalyzed reaction between CoA and CoA-M in water was monitored by long-time fluorescence measurements using a Perkin-Elmer LS55 spectrofluorometer, see Supplementary Figure **3**. Experiments were initiated by mixing 1.5 ml of 100 μM aqueous solution of each reactant in a quartz cuvette with a path length of 10 mm for excitation and 10 mm for emission. The excitation wavelength was set to 390 nm, and emission was monitored at 550 nm, with both excitation and emission band-pass set to 2.5 nm. The integration time per data point was 0.5 s. Each data point was collected every 15 min. The fluorescence was measured over 8 days and allowed us to estimate the bimolecular reaction rate constant as 0.05 $M^{-1}s^{-1}$.

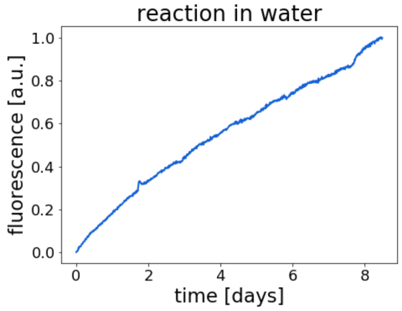

Supplementary Figure 3: Reaction between CoA and CoA-M in water, T = 25 ℃.

Supplementary Note 5. Estimation of the critical micelle concentration.
We estimated critical micelle concentration (CMC) for cetrimonium chloride (CTAC) in water and MES buffer utilizing a volume-based thermodynamics model elaborated by Preiss Ulrich P., et al. [1] The model can predict a CMC of ionic surfactants, including ionic liquids, with added salts. To calculate the CMC of CTAC, we had to determine prior its solvent-accessible surface area, $\hat{S}$, free solvation enthalpy, $\Delta_{solv}G$, molecular volume, V_m, as well as zwitterion's molecular volume, $V_{m,z}$. The solvent-accessible surface area was calculated with PyMOL software, [2] free solvation enthalpy with Conductor-like Screening Model, [3] and the molecular volume of surfactant and zwitterion according to the ref. [4] The obtained parameters were as follows: $\hat{S}$ = 4.88 nm^2, $\Delta_{solv}G$ = -558 kJ·mol^{-1}, V_m = 0.33 nm^3, $V_{m,z}$ = 0.15 nm^3 The calculated CMC of CTAC in water was 1.06 mM, in 1 mM phosphate buffer was 1.05 mM and in 50 mM MES was 0.70 mM. The CMC of CTAC in MES is lower than the one in water due to the decrease in the columbic repulsion of the head groups, which results from the screening of the charge on the CTAC.

Since we didn't perform measurements for other surfactants in MES, we took CMC values in water for cetylpyridinium chloride, benzethonium chloride, sodium dodecyl sulfate, and Brij L23 from references.

Supplementary Note 6. Influence of pH on the catalysis.
We measured whether a change in pH in the presence of polylysine or cetrimonium chloride influences the reaction rate. Both the CoA and CoA-M concentration was 10 μM, the concentration of CTAC was 1.35 mM, and the concentration of polylysine was $\sim$0.6 μM. As one can see from Supplementary Figure **4**, increasing pH accelerates the reaction, both in the case of polylysine and CTAC. Partially it is due to the increase in ionic strength since to increase the pH of the solution, we added sodium hydroxide. As in the case of sodium chloride (see Main Article Figure 2a), sodium ions screen the negative charge of the reactants. However, a more significant impact on acceleration is contributed to the loss of hydrogen ion by bromo-N-methylmaleimide moiety. By introducing hydroxyl ions into the solution, the thiol group present in the fluorophore loses hydrogen and turns into a thiolate. As a result, its nucleophilic character increases, and the fluorescent reactant binds easier to the non-fluorescent reactant.

For all the conditions presented in this study, we assumed that the charge of the reactants (CoA and CoA-M) is -4. However, pK_a of the secondary ionization of the phosphate group in CoA is 6.4. [5,6] Thus, in MES of pH 6.1 with no additional co-solutes, the reactants' charge could be in the form of -4 and -3. Nevertheless, when the reaction was studied in MES, reactants were co-soluted with positively charged catalysts that shift phosphate pK_a significantly towards the lower values, and CoA exists mainly in the form of charge -4. [7]

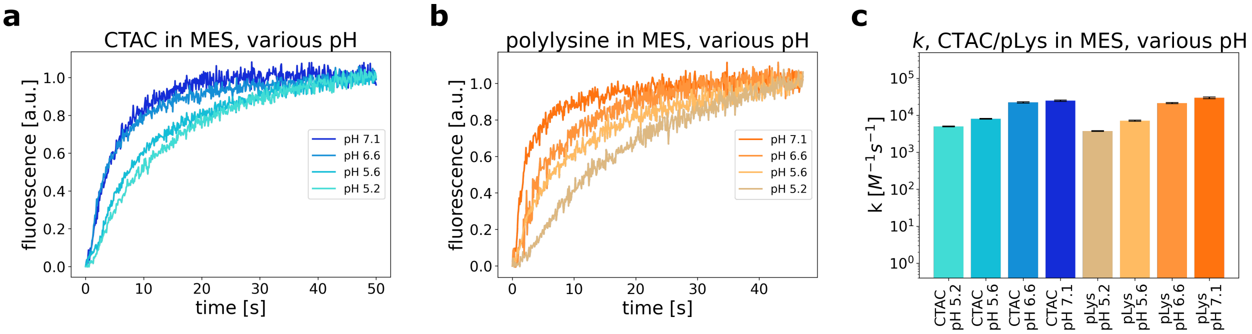

Supplementary Figure 4: Influence of pH on the reaction acceleration in 50 mM MES in the presence of **(a)** CTAC, **(b)** polylysine, and **(c)** theirs reaction rate constants. Error bars for 4c correspond to weighted mean errors calculated as described in Supplementary Note 3.

Supplementary Note 7. Reaction at the glass-solution interface

We observed that adding cationic surfactants at few μM concentrations (much lower than CMC) does not speed up the Coenzyme A reaction when measuring in bulk (15-20 μm from the measuring cell bottom), which depicts Supplementary Figure **5**a. Because surfactants first saturate the glass-solution interface and create a layer therein before the appearance of free surfactants and micelles (when higher concentration is used) in bulk. [8] Therefore, we analyzed what is happening at the glass-solution interface. Using a confocal microscope that allows collecting fluorescent intensity from the femtoliter volumes, we were able to see these particular parts of the entire sample. As shown for the reaction in the presence of 6 μM CTAC in Supplementary Figure **5**b, the signal at the glass surface significantly varies what represents reaction progress. We confirmed catalytic properties of positively charged surfactants at glass surface by performing control measurement with 6 μM of neutral surfactant (Brij). The result is presented in Figure **5**c, and shows no occurrence of the reaction.

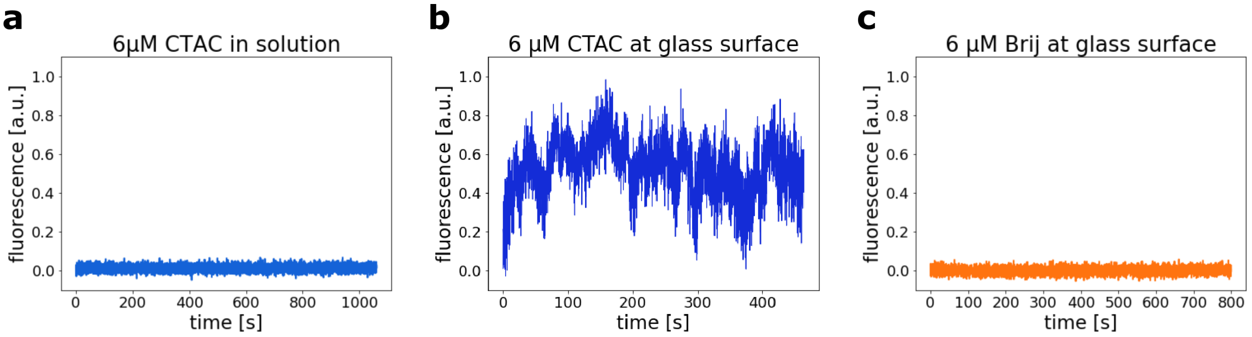

Supplementary Figure 5: The reaction of CoA and CoA-M in the presence of 6 μM CTAC (below CMC) **(a)** in the bulk solution and **(b** at the glass surface; **(c)** reaction in the presence of 6 μM Brij (below CMC) at the glass surface. T = 25 °C.

Supplementary Note 8. Monitoring DNA hybridization by FRET.
We monitored the kinetics of DNA-DNA double-strand complex formation by utilizing FRET. Photons emitted from the donor (Atto 488) and acceptor (Atto 647N) were registered separately in two channels. In Channel 1, photons originated from the acceptor (645 nm long-pass filter), and in Channel 2, from the donor (525/50 nm band-pass filter). We set the concentration of oligonucleotides to 10 nM and added 0.002 % TWEEN 20 to prevent reactants from accumulating on the glass surface.

The electrostatic repulsion of the complementary strands prevents the formation of a double-stranded form (complex) in water due to the lack of screening ions in the buffer. Even after 14 days, the equilibrium of the reaction is indistinguishable from the initial state, see Supplementary Figure **6**a,b,c. Performing following experiment in weak buffer (1 mM PB) allowed us to determine association constant $k = 2.69 \cdot 10^4$ $M^{-1}s^{-1}$, shown in Supplementary Figure **6**d.

To reduce repulsion forces between negatively charged DNA backbones, we introduced various positively charged catalysts. In Supplementary Figure **6**d,e increase of association rate by ~500 times (in comparison with 1 mM PB buffer only) is observed when applying surfactants at CMC.

To show that the influence of surfactants is purely charged-based, we performed experiments on the negatively charged micelles (SDS) and micelles without charge (Brij), Supplementary Figure **6**f. Results show no complex formation.

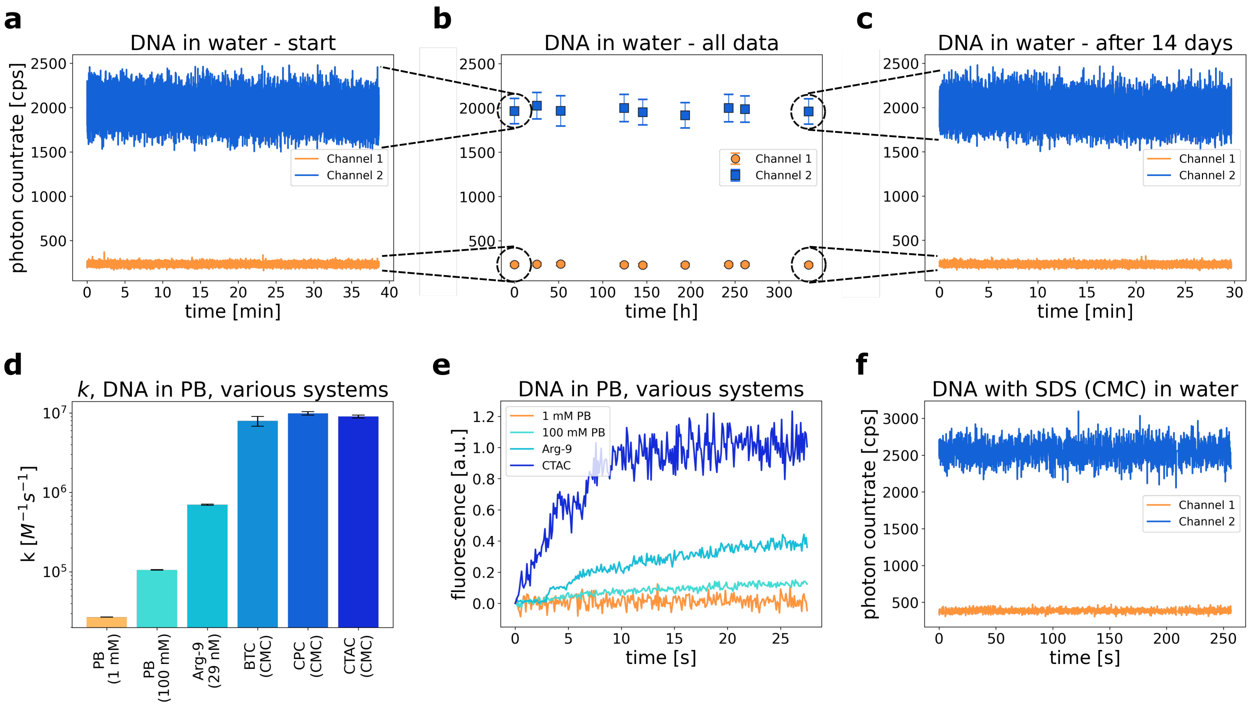

Supplementary Figure 6: **(a)** Time trace signal registered at the beginning of the measurement, and **(c)** after 14 days. **(b)** Reaction between DNA-DNA single strands in water, T = 25 ℃ presented as an average signal. **(d)** Reaction rate constants of DNA double-strand formation in the presence of various catalysts in 1 mM PB buffer, T = 25℃, and **(e)** examples of time trace signal used to calculate association constant.**(e)** Reaction between DNA-DNA single strands with SDS, in water with 0.002 % TWEEN 20. Surfactant concentrations is equal to its CMC value. Error bars for 6b correspond to standard deviation, whereas for 6d correspond to weighted mean errors calculated as described in Supplementary Note 3.

Supplementary Note 9. Modeling effect of Coulomb interactions on reaction kinetics.

Alike charged reactants rarely meet on their own.

We discuss the generic speedup mechanism using the example of the CoA. The structure of a single coenzyme is close to linear, which extends over $l \approx 2$ nm with a reaction centre at one end and a charge of $-4e$ localized approximately in the middle. Constant Brownian rotation causes the drift of the reaction centre over the sphere of radius 1 nm around the charge. For a rigid rod having a length of 2 nm and 1 nm thickness (in water at 293.15 K), a typical time over which a rod's orientation decorrelates is roughly 4×10^{-10} s, and the diffusion time on the length scale of its length is 5×10^{-9} s [9]. Therefore, we approximate it as a sphere with a reacting surface of radius $l/2 = 1$ nm with a charge localized in its centre. In such a view, the irreversible reaction between two coenzymes, which in reality occurs when their reaction centres meet, happens when the reacting surfaces touch each other. Equivalently, a reaction occurs when charges localized in the centre of the coenzyme reach the proximity of l.

According to Smoluchowski's approach [10, 11], the chance of finding another particle around a chosen particle is given by the pair distribution function P_2. The Smoluchowski equation describes the high viscosity (over-damped) limit of Brownian motion in a force field [12]. In spherical coordinates, with spherically symmetric interaction potentials and in a stationary state, P_2 can be calculated from the following equation

$$\frac{1}{r^2}\frac{\mathrm{d}}{\mathrm{d}r}r^2 D\left(P_2\frac{\mathrm{d}}{\mathrm{d}r}\frac{\Phi}{k_BT} - \frac{\mathrm{d}}{\mathrm{d}r}P_2\right) = 0, \tag{8}$$

where Φ is interaction potential, k_BT is the energy of thermal motion, $D = (D_1 + D_2)$ and $D_{1,2}$ are diffusion coefficients of particle $1, 2$ in the direction of the connector vector between particle centres. The boundary conditions are $P_2(l) = 0$ at the reaction surface and $P_2(\infty) = 1$ away from the reaction surface, which means that particles react immediately upon contact and there is no disturbance of bulk distribution far away from the chosen particle. For the sake of simplicity, we assume that hydrodynamic interactions do not play here a significant role and therefore $D_{1,2}$ are constant. Such an assumption is, in general, an oversimplification. However, we focus on the influence of the particle interaction potential, which appears to be more important as it enters the theory through the exponential function while space varying diffusion through a multiplicative factor. From the pair distribution function, one can calculate the total pair flux through the reaction surface

$$J_2 = 4\pi\left(\frac{l}{2}\right)^2 D\left(P_2\frac{\mathrm{d}}{\mathrm{d}r}\frac{\Phi}{k_BT} - \frac{\mathrm{d}}{\mathrm{d}r}P_2\right) = \frac{\pi D l^2}{\int_l^\infty \frac{\exp\left(\frac{\Phi(r)-\Phi(\infty)}{k_BT}\right)}{r^2}\mathrm{d}r}. \tag{9}$$

It is a central quantity in our reasoning. From J_2, the total matter flux through the reacting surface is obtained through the multiplication by the number densities of reactants in undisturbed condtinions $J = J_2 n_1 n_2$. The last quantity can be interpreted as the reaction rate according to the equation

$$\frac{\mathrm{d}n_1}{\mathrm{d}t} = \frac{\mathrm{d}n_2}{\mathrm{d}t} = -J = -J_2 n_1 n_2. \tag{10}$$

First, we consider two model situations (Supplementary Table **1**): i) spherical particles without interactions, and ii) spherical particles carrying point electric charges q_1, q_2 in their centers. We neglect the presence of the image charges that appear when there is a difference in the dielectric constants, which means that we assume that the dielectric constant of water is the same as of that of suspended particles [13].

Supplementary Table 1: Potentials and associated pair flux rates. In the above $\kappa = \frac{q_1 q_2}{4\pi\varepsilon_0\varepsilon k_BTl}$ is the dimensionless energy scale.

case	i)	ii)
potential	$\Phi = 0$	$\Phi = \kappa\frac{l}{r}$
pair flux	$J_2 = \pi D l$	$J_2 = \pi D l \frac{\kappa}{e^\kappa - 1}$

The associated pair fluxes can be calculated analytically (Supplementary Table **1**). Case i) is a reference value when no interactions are present and is a limit of cases ii) when $\kappa \to 0$. In case ii) J_2 is exponentially

decaying for large positive κ that is, for the same sign charges. For the opposite sign charges (negative κ), J_2 grows linearly. For the proposed CoA model at $T = 300$ K we calculate $\kappa \approx 5.7$, which strongly depends on the choice of l.

Screening effect of ions.

The addition of freely diffusing ions in the system introduces the effect of charge screening due to the buildup of electrical double layers at the surface of particles. The Debye lenght, which determines the lenght scale over which the electric field decays is $\lambda_D = \left(\frac{\varepsilon_0 \varepsilon k_B T}{e^2 \sum_i n_i}\right)^{1/2}$. For 50 mM concentration of NaCl in water (as in experiments) $\lambda_D \approx 1.4$ nm, for 200 mM $\lambda_D \approx 0.7$ nm, for 50 mM MES $\lambda_D \approx 2.4$ nm, and for 1 mM Phosphate Buffer $\lambda_D \approx 6.6$ nm. As a result, the ratio between reaction rates in pure water and in water with 50 mM NaCl is $0.054/12.9 \approx 0.004$, and in pure water and in water with 200 mM NaCl is $0.054/20.7 \approx 0.003$. The predicted ratio of J_2 between unscreened and completely screened interactions (cases i) and ii)) is $\frac{5.7}{\exp(5.7)-1} \approx 0.019$. For assumed l, we interpret that 50 mM salt concentration is already close to complete screening of electrostatic interactions $\lambda_D \leq l$. However, they are not entirely screened as adding further salt increases the reaction rate. We understand that this discrepancy comes from our approximate assumptions.

The influence of a large oppositely charged catalyst.

Large, positively charged particles introduce another length scale to the problem: a typical radius of a catalyst R. Often $\lambda_D < R$, which suggests that reactants interact electrostatically only with an element of the catalyst's surface. Therefore, we expect that a major role is played by the surface charge density of the catalyst

$$\chi = q_c/S_c, \tag{11}$$

where q_c is the charge of the catalyst, and S_c is the surface area of the catalyst. One can calculate S_c, e.g., as a surface area of a 3D model of a molecule that is accessible by a spherical probe. In this contribution, we used a probe with 1.4 Å radius, which is typical for determining water accessible area.

The Debye-Hückel model of the electrical double layer above the flat surface gives the electric potential $\Psi(x) = \Psi_D e^{-\frac{x}{\lambda_D}}$, where Ψ_D is the electric potential at the surface, and x is the distance from the surface [14]. Assuming $\Psi(\infty) = 0$ and knowing the surface ($x = 0$) charge density χ we can determine that $\Psi_D = \frac{\chi \lambda_D}{2\pi\varepsilon_0\varepsilon}$. Consequently, we can determine the potential energy of a reactant charge q_s and the surface

$$\Phi(x) = \frac{q_s \chi \lambda_D}{2\pi\varepsilon_0\varepsilon} e^{-\frac{x}{\lambda_D}}. \tag{12}$$

It reveals a dimensionless energy scale

$$\kappa = \frac{\Phi(0) - \Phi(\infty)}{k_B T} = \frac{q_s \chi \lambda_D}{2\pi\varepsilon_0\varepsilon k_B T}. \tag{13}$$

For example, for a pair Arg-9 and CoA in 50 mM NaCl $\kappa \approx -113$, and the same pair in 50 mM MES $\kappa \approx -208$.

Next, we consider a spherical reactant with diffusion coefficient D undergoing Brownian motion in the potential (**12**), near the reacting surface of area A. It is possible to formally solve the Smoluchowski equation in Cartesian coordinates in 1D

$$\frac{\mathrm{d}}{\mathrm{d}x} D \left(P_2 \frac{\mathrm{d}}{\mathrm{d}x} \frac{\Phi}{k_B T} - \frac{\mathrm{d}}{\mathrm{d}x} P_2 \right) = 0, \tag{14}$$

with boundary conditions $P_2(0) = 0$ and $P_2(L) = 1$. The latter means that the reservoir of reactants is at a distance L and typically $L > \lambda_D$. We find

$$J_2 = AD \left(P_2 \frac{\mathrm{d}}{\mathrm{d}x} \frac{\Phi}{k_B T} - \frac{\mathrm{d}}{\mathrm{d}x} P_2 \right) = \frac{AD}{\int_0^L \exp\left(\kappa \left(\exp(\frac{x}{\lambda_D}) - \exp(\frac{L}{\lambda_D})\right)\right) \mathrm{d}x}. \tag{15}$$

Luckily, in the limit of $\kappa \ll -1$, the flux can be analyzed analytically by series expansion, and we find

$$J_2 \approx -J_2^0 \kappa, \tag{16}$$

where $J_2^0 = \frac{DA}{\lambda_D} e^{-L/\lambda_D}$ is a positive proportionality constant. This result is similar to that of two oppositely charged particles yet with a differently defined energy scale κ. J_2 and J_2^0 in suitable units can be interpreted as Smoluchowski reaction rate k^S and k_0^S, respectively.

We do not calculate the proportionality constant J_2^0 for each reactant-catalyst pair from the first principles as there are too many unknowns. For instance, it is unclear what should be chosen as L for every type of reactants or external conditions like mixing. Moreover, measured reaction rates strongly depend on the details of reactant chemistry. Nevertheless, we can calculate the diffusive relaxation time on the Deblye lenght $\tau_{\lambda_D} = \lambda_D^2/D$, which for CoA and 50 mM NaCl (and 50 mM MES) concetration is on the order of 10^{-9} s. The magnitude of τ_{λ_D} suggests that J_2 will set the concentration of reactants at the surface of the catalyst immediately in comparison to other processes. We hypothesize that J_2 directly influences the reaction rate by elevation of reactant collision frequency. In result, we write that for each reactant type the reaction rate

$$k = -k_0 \kappa. \tag{17}$$

Here, κ involves χ, which changes for each catalyst and q_s, which changes for each reactant. From multiple catalyst-reactant pairs measurements, we can determine k_0 by fitting a line. We plot reactant normalized k/k_0 reaction rate as a function of reactant-catalyst pair -κ. The results for CoA and DNA (Manuscript Figure 6) reveal that the catalytic activity is well explained by the electrostatic increase of reactant flux towards the surface of the catalyst. Finally, the multiplicative character of the reaction rate increase supports the hypothesis that the speed up happens due to the locally increasing encounters of reactants at the surface of the catalytic particle.

The influence of the catalyst concentration.

The systematic study of the reaction rate (Figure 4e) as a function of catalyst concentration shows a maximum, which we explain with the help of the two-step mechanism model. The first step is the transport of the free reactant particles s^{bulk} to the surface of the catalyst c and the formation of a complex s^{cat}. We interpret it as an irreversible second-order reaction

$$s^{\text{bulk}} + c \xrightarrow{k_1} s^{\text{cat}}. \tag{18}$$

The reaction rate

$$k_1 \propto s^{\text{bulk}} c, \tag{19}$$

where s^{bulk} is the concentration of bulk reactant and c is the concentration of the catalyst. The second step is the reaction between two reactants that are at the surface of the catalyst, which yields the product p

$$s^{\text{cat}} + s^{\text{cat}} \xrightarrow{k_2} p. \tag{20}$$

Here, the reaction rate $k_2 \propto k^{\text{cat}} \left(\sigma^{\text{cat}}\right)^2$, where k^{cat} is the reaction rate between two reactants at the surface of the catalyst and $\sigma^{\text{cat}} = \frac{s^{\text{cat}}}{c S_c \lambda}$ is an effective concentration of the reactant at the surface of the catalyst. In the proposed formula, λ is an effective thickness over the surface of catalyst S_c, which is occupied by the reactant in complex with the catalyst. Therefore, we can write that

$$k_2 \propto k^{\text{cat}} \left(\frac{s^{\text{cat}}}{c S_c \lambda} \right)^2. \tag{21}$$

The final reaction rate is limited by a minimum of k_1 and k_2, which have a different dependence on c. Asymptotically, for low c the reaction rate is limmited by k_1, which is linearly growing as a function of c and at the same time $k_1 \ll k_2$. For large c the reaction rate will be limited by k_2 as it decreases as c^{-2} and $k_2 \ll k_1$. This asymptotic behaviour is true regardless of other parameters in the system. Therefore, we expect that an optimal catalyst concentration exists, and it depends, among others, on the details of reactant reactions at the catalyst's surface. Indeed, in the Supplementary Figure 4e we observe three distinct regimes. First, between 0.1 and 0.2 μM concentrations of CTAC micelles there is an increase in the measured k. Second, between 0.2 and 50 μM concnentrations there is a systematic decrease in measured k. Dilution of the catalyst by 250 times changes the reaction rate approximately 10 times, which we interpret as weak variation. It is surprising and constitutes another premise to neglect the dependence of reaction rate on the

catalyst concentration and focus on κ as a main actor instead. Third, between 50 and 100 μM concentration there is a drop of k by more than 10 times. The 100 μM concentration of CTAC micelles means that there are 10 micelles per 1 reactant molecule, which suggests that reactants rarely populate the same catalyst. This shows that the reaction rate increase is significantly hindered by adding much more catalyst particles than reactants.

Supplementary Table 2: Summary of reaction rates and following experimental conditions for Coenzyme-A reaction model.

**CoA and CoA-M reaction**						
system	pH	$C_{reactant}$	catalyst	$C_{catalyst}$	k [$M^{-1}s^{-1}$]	error [%]
water	7	10 μM	—	—	$5.40 \cdot 10^{-2}$	3.70
			Arg9	8.9 μM	$6.42 \cdot 10^{2}$	0.61
			CTAC	0.1 μM micelle	$1.66 \cdot 10^{5}$	12.33
			CTAC	0.2 μM micelle	$2.75 \cdot 10^{5}$	12.81
			CTAC	0.5 μM micelle	$9.77 \cdot 10^{4}$	12.53
			CTAC	1 μM micelle	$3.71 \cdot 10^{4}$	5.56
			CTAC	5 μM micelle	$2.83 \cdot 10^{4}$	11.59
			CTAC	10 μM micelle	$2.37 \cdot 10^{4}$	7.70
			CTAC	50 μM micelle	$1.49 \cdot 10^{4}$	6.1
			CTAC	100 μM micelle	$1.12 \cdot 10^{3}$	1.25
			CTAC	1.35 mM	$2.28 \cdot 10^{4}$	4.23
		20 μM	CTAC	20 μM micelle	$2.91 \cdot 10^{3}$	2.57
			CPC	20 μM micelle	$2.95 \cdot 10^{3}$	3.28
			BTC	20 μM micelle	$7.68 \cdot 10^{2}$	1.87
			SDS	20 μM micelle	—	—
			Brij	20 μM micelle	—	—
50 mM MES	5.2	10 μM	pLys	0.6 μM	$3.76 \cdot 10^{3}$	1.91
			CTAC	1.35 mM	$5.03 \cdot 10^{3}$	2.27
	5.6	10 μM	pLys	0.6 μM	$7.13 \cdot 10^{3}$	3.49
			CTAC	1.35 mM	$8.06 \cdot 10^{3}$	2.12
	6.1	10 μM	—	—	$2.27 \cdot 10$	0.40
			pLys	0.6 μM	$1.24 \cdot 10^{4}$	2.49
			Arg	80 μM	$4.32 \cdot 10$	0.86
			Arg9	8.9 μM	$6.77 \cdot 10^{2}$	0.61
			CTAC	0.1 mM	$2.61 \cdot 10$	0.61
			CTAC	1.1 mM	$1.92 \cdot 10^{4}$	3.53
			CTAC	1.35 mM	$1.72 \cdot 10^{4}$	3.03
			CTAC	3.5 mM	$2.35 \cdot 10^{2}$	0.54
			CTAC	10 mM	$3.12 \cdot 10$	1.47
			CPC	~CMC	$2.48 \cdot 10^{4}$	4.61
			BTC	~CMC	$4.59 \cdot 10^{3}$	1.97
	6.6	10 μM	pLys	0.6 μM	$2.14 \cdot 10^{4}$	3.55
			CTAC	1.35 mM	$2.24 \cdot 10^{4}$	3.83
	7.1	10 μM	pLys	0.6 μM	$2.99 \cdot 10^{4}$	5.50
			CTAC	1.35 mM	$2.49 \cdot 10^{4}$	4.08
200 mM MES	6.1	10 μM	—	—	$3.48 \cdot 10$	0.49
			Arg9	8.9 μM	$4.27 \cdot 10^{2}$	0.60
			CTAC	1.35 mM	$5.10 \cdot 10^{3}$	1.42
500 mM MES	6.1	10 μM	—	—	$3.90 \cdot 10$	0.59
			Arg9	8.9 μM	$2.92 \cdot 10^{2}$	0.61
			CTAC	1.35 mM	$5.99 \cdot 10$	0.25
50 mM NaCl	7	10 μM	—	—	$1.29 \cdot 10$	0.39
200 mM NaCl	7	10 μM	—	—	$2.07 \cdot 10$	0.39
500 mM NaCl	7	10 μM	—	—	$2.67 \cdot 10$	0.45

Supplementary Table 3: Summary of reaction rates and following experimental conditions for DNA hybridization.

**DNA hybridization**						
system	pH	$C_{reactant}$	catalyst	$C_{catalyst}$	k [$M^{-1}s^{-1}$]	error [%]
water	7	10 nM	—	—	—	—
			Arg9	29 nM	$1.23 \cdot 10^6$	1.48
			CTAC	∼CMC	$1.01 \cdot 10^7$	11.91
			CPC	∼CMC	$9.33 \cdot 10^6$	5.45
			BTC	∼CMC	$3.44 \cdot 10^6$	2.07
			SDS	∼CMC	—	—
			Brij	∼CMC	—	—
1 mM PB	7.4	10 nM	—	—	$2.70 \cdot 10^4$	0.23
			Arg9	29 nM	$7.02 \cdot 10^5$	1.53
			CTAC	∼CMC	$9.04 \cdot 10^6$	4.32
			CPC	∼CMC	$9.88 \cdot 10^6$	4.95
			BTC	∼CMC	$7.94 \cdot 10^6$	14.07
100 mM PB	7.4	10 nM	—	—	$1.06 \cdot 10^5$	0.84

Supplementary References

1. Ulrich P Preiss, Philipp Eiden, Justyna Łuczak, and Christian Jungnickel. Modeling the influence of salts on the critical micelle concentration of ionic surfactants. *Journal of colloid and interface science*, 412:13–16, 2013.

2. Warren L DeLano et al. Pymol: An open-source molecular graphics tool. *CCP4 Newsletter on protein crystallography*, 40(1):82–92, 2002.

3. Andreas Klamt and GJGJ Schüürmann. Cosmo: a new approach to dielectric screening in solvents with explicit expressions for the screening energy and its gradient. *Journal of the Chemical Society, Perkin Transactions 2*, 2(5):799–805, 1993.

4. Bhyravabhotla Jayaram, Tanya Singh, Goutam Mukherjee, Abhinav Mathur, Shashank Shekhar, and Vandana Shekhar. Sanjeevini: a freely accessible web-server for target directed lead molecule discovery. *BMC bioinformatics*, 13(17):1–13, 2012.

5. David A Keire, Jan M Robert, and Dallas L Rabenstein. Microscopic protonation equilibria and solution conformations of coenzyme a and coenzyme a disulfides. *The Journal of Organic Chemistry*, 57(16):4427–4431, 1992.

6. Helmut Beinert, RW Von Korff, DE Green, DA Buyske, RE Handschumacher, Harvey Higgins, and FM Strong. A method for the purification of coenzyme a from yeast. *Journal of Biological Chemistry*, 200(1):385–400, 1953.

7. D Eric Anderson, Wayne J Becktel, and Frederick W Dahlquist. ph-induced denaturation of proteins: a single salt bridge contributes 3-5 kcal/mol to the free energy of folding of t4 lysozyme. *Biochemistry*, 29(9):2403–2408, 1990.

8. Krzysztof Bielec, Krzysztof Sozanski, Marco Seynen, Zofia Dziekan, Pieter Rein Ten Wolde, and Robert Holyst. Kinetics and equilibrium constants of oligonucleotides at low concentrations. hybridization and melting study. *Physical Chemistry Chemical Physics*, 21(20):10798–10807, 2019.

9. Pawel J Zuk, Bogdan Cichocki, and Piotr Szymczak. Grpy: an accurate bead method for calculation of hydrodynamic properties of rigid biomacromolecules. *Biophysical journal*, 115(5):782–800, 2018.

10. Marian von Smoluchowski. Versuch einer mathematischen theorie der koagulationskinetik kolloider lösungen. *Zeitschrift für physikalische Chemie*, 92(1):129–168, 1918.

11. William Bailey Russel, WB Russel, Dudley A Saville, and William Raymond Schowalter. *Colloidal dispersions.* Cambridge university press, Cambridge, 1991.

12. Hendrik Anthony Kramers. Brownian motion in a field of force and the diffusion model of chemical reactions. *Physica*, 7(4):284–304, 1940.

13. Paweł Jan Żuk. A brief introduction to contemporary electrokinetics. *Contemporary Physics*, pages 1–13, 2022.

14. Jacob N Israelachvili. *Intermolecular and surface forces.* Academic press, San Diego, 2011.
